# Metric evaluation of the anterior nasal spine to estimate sex and population group in South African individuals

**DOI:** 10.1007/s00414-023-03130-x

**Published:** 2023-11-27

**Authors:** Christy Lana Davidson, Johan de Klerk, Zina Matejovsky, Inger Fabris-Rotelli, Andre Uys

**Affiliations:** 1 Department of Oral and Maxillofacial Pathology, School of Dentistry, Faculty of Health Sciences, University of Pretoria, PO Box 1266, Pretoria, 0001 South Africa; 2 Department of Statistics, Faculty of Natural and Agricultural Sciences, University of Pretoria, Pretoria, South Africa; 3 Department of Anatomy, School of Medicine, Faculty of Health Sciences, University of Pretoria, Pretoria, South Africa

**Keywords:** Anterior nasal spine, Cone beam computed tomography, Decision tree

## Abstract

**Introduction:**

The anterior nasal spine is a pointed, midline projection of the maxilla. This bony structure dictates the overlying soft tissues providing the phenotypic features of the nose and upper lip and determines the differences in the mid-face morphology. Little data is available on the metric features of the Anterior nasal spine (ANS). This study aimed to perform metric evaluations of the ANS of white and black South African males and females to ascertain if morphological variations exist and if the differences are viable for the use in sex and population identification.

**Materials and methods:**

The sample included 100 CBCT images for each population and sex group. Linear and angular measurements of the ANS were recorded in both the sagittal and axial planes.

**Results:**

Classification decision trees (pruned) were fitted to ascertain the relationship between population group, sex and the ANS measurements including and excluding age.

For population group, all the ANS measurements were statistically significant for females but in males, all the ANS measurements were significant when performed individually. However, when fitted to the classification tree, Sagittal 2 did not show any statistical significance. When considering sex, only 2 of the ANS measurements (Sagittal 2 and Axial 1) were found to be significant. The results did not differ significantly when comparing the decision trees including and excluding age.

**Conclusions:**

White South African individuals presented with a longer ANS that produced a more acute angle whereas black South African individuals presented with a shorter ANS and a more obtuse angle. Additionally, males presented with a longer ANS compared to females. ANS measurements were found to be more relevant for population discernment than for sex.

## Introduction

The anterior nasal spine (ANS) is a pointed, midline projection of the maxilla situated inferior to the nasal cartilages. This bony structure dictates the overlying soft tissue morphology providing the phenotypic features of the nose and upper lip [1, 2]. The midface, especially the nasal region, is considered to have traits valuable in distinguishing population origin [1]. The ANS is a macromorphoscopic trait that can be used in conjunction with cranial metrics to determine population [3]. It is also a commonly used landmark during cephalometric radiological projections [4].

Anatomical variation of the ANS exists between different populations. Three categories have been defined: Asian, African and European [5–9]. In the European group, the ANS is described as pronounced and straight with a long anterior projection. In the Asian group, it is found to be “medium” or “moderate” and in the African group reduced or even absent. These characteristic morphological variations are established early in the fetal development and are maintained postnatally [10]. Already at 24 weeks prenatal, European specimens present with a much more prominent ANS when compared to African specimens, who in turn show a much more reduced ANS at the same developmental stage [10]. Further support of the morphological differences was demonstrated in a study comparing black and white perinates where the white perinates exhibited a more prominent ANS compared to their black counterparts at the same stage [11]. However, in a South African study, these distinct variations were proven to partially disappear with age, with the ANS more often classified as long and prominent with advanced age. This finding was evident regardless of population group [12]. Nasofacial growth was shown to peak before 16 years of age in females and 17 years of age in males [13].

Sex determination is critical in the identification of missing and unidentified individuals as well as in the search for suspects or victims of crime [14]. Morphological differences in features between males and females are commonly accepted with many studies having evaluated these differences [15–23]. Furthermore, a craniomorphometric analysis of skulls showed that a few cranial measurements were significant for sex determination and some appear to be independent from population [24]. Studies on population-specific sex variations are critical as differences can occur between population groups. A study investigating the sexual dimorphism patterns of South African male and female crania demonstrated that South Africans showed less sexual dimorphism when compared to a Northern American population [15]. White South African individuals show larger differences between the sexes in contrast to Black South African males and females [25].

Non-metric cranial traits are morphological features that occur in the cranium. These traits are considered informative in distinguishing between different sexes and populations. It is important to remember that no single trait can be used in isolation for identification and as such, ANS features should be used in conjunction with other cranial and facial landmarks [4, 7]. Hefner identified ten traits that were significant in determining population origin, of which the ANS was one [7]. However, more than one study has shown a large amount of within-observer variation when examining these traits [7, 12]. A shortcoming of prior evaluations of the ANS is that they are non-specific, often only distinguishing between “short”, “medium”, and “long” or slight, intermediate and marked nasal spines. This creates an experience-based classification system that is not reproducible and therefore inconsistent for use in population identification [7]. Furthermore, the lack of research regarding ANS variation among non-racial-based variables renders the ANS of little forensic use outside of a population context. Hence, metric evaluations of ANS variation and studies investigating this variation in sex and age are required.

An understanding of ANS variation has significant potential in the field of forensic sciences to aid in the identification of deceased individuals. It offers to distinguish between (and subsequently exclude) very large population groups for consideration during the identification process. As a bony structure, it decomposes at a much slower rate than soft tissues and will retain its shape for many years after death despite losing structural integrity over time. It can therefore be useful by virtue of usually being present in the post-mortem setting.

The aim of this study was to perform metric evaluations of the ANS of individuals from different population and sex groups on cone-beam computed tomography (CBCT) images in a South African sample and to ascertain if morphological variations existed between them.

## Materials and methods

Cone-beam computed tomography images of 400 individuals of known age and sex were selected from the archives of the Diagnostic Imaging Unit, School of Dentistry, University of Pretoria, South Africa. This included 100 CBCT images for each black (BSA), white (WSA), male (MSA) and female (FSA) group. The radiographs were selected using a convenience sampling method. Population group was self-classified according to the patient’s hospital file. All CBCT images used were part of the patient’s routine dental treatment and no CBCT scans were taken primarily for this study. Approval to conduct the study was obtained from the Faculty of Health Sciences Research and Ethics Committee of the University of Pretoria (417/2020).

The CBCT images required the following criteria for inclusion in the study:

- Adequate demographic information regarding the population group, sex and age of the patient.
- Individuals that were older than 22 years of age, as this indicated that growth had ceased.
- The anatomical area of ANS had to be included on the scan and clearly visible.

The following CBCT images were excluded from the study:

- Presence of any pathology, surgery or growth disturbance in the maxillary region.

### Measurements

The metric data of the ANS was evaluated on the CBCT images in both the sagittal and axial planes where the ANS was most pronounced. Both linear and angular measurements were recorded.

**Fig. 1:**
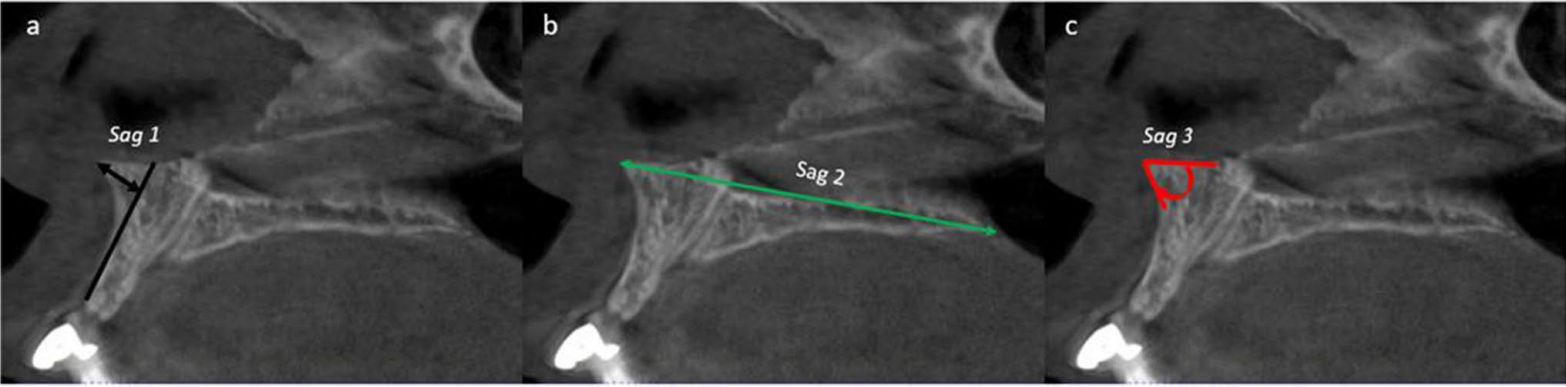
Measurements of the ANS taken in the sagittal plane (*Sag1, Sag2, Sag3)*

In the sagittal plane (Fig. 1):

**• Sagittal 1 (*Sag1*):** length (mm) from the most anterior point of the ANS to the point on a perpendicular line running along the anterior cortical plate of the maxilla.
**• Sagittal 2 (*Sag2*):** length (mm) from the most anterior point of the ANS to the most posterior point of the posterior nasal spine (i.e. the distance of the nasal floor).
**• Sagittal 3 (*Sag3*):** The angle created from the two lines extending from the most anterior point of the ANS that intersected a line following the anterior maxillary cortical plate.

**Fig. 2:**
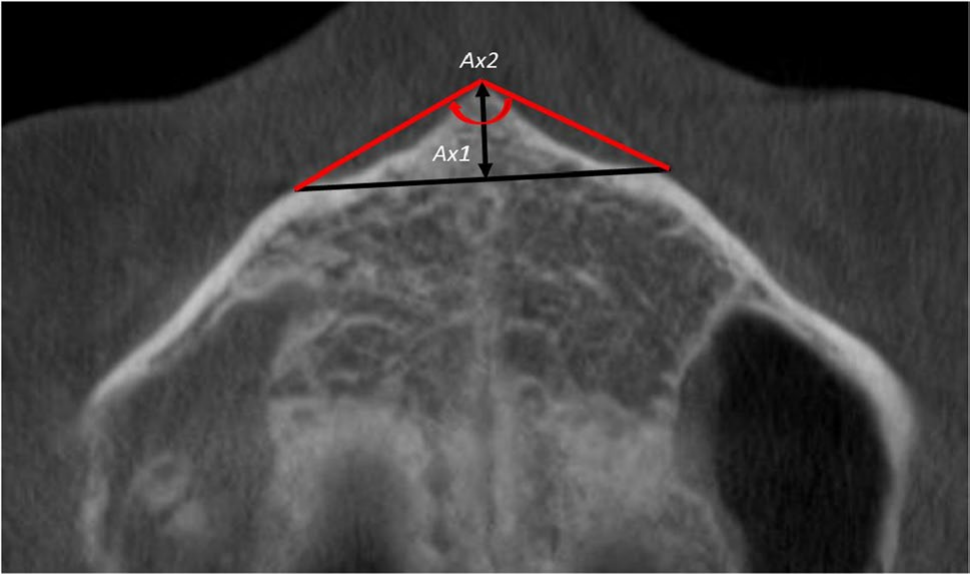
Measurements of the ANS taken in the axial plane (*Ax1* and *Ax2*)

For the collection of the ANS data in the axial plane, the CBCT image was positioned in the sagittal plane where a line from the most anterior point of the ANS lay perpendicular to a line running along the anterior maxillary cortical plate. This allowed a consistent method of assessment in the axial plane. Once the image had been positioned, the following measurements were collected in the axial plane (Fig. 2):

**• Axial 1 (*Ax1*):** length (mm) from the most anterior point of the ANS to a line drawn perpendicular to the line connecting the most pronounced area of the canine bulges.
**• Axial 2 (*Ax2*):** angle formed from the most anterior aspect of the ANS to the cortex of the most pronounced area of the canine bulges.

The measurements of the ANS were collected by two examiners independently. Thereafter the CBCT images were randomised and re-evaluated. In instances where the examiners noted discrepancy in their measurements, these measurements were highlighted and these cases were re-measured by the examiners together until there was agreement in the measurements. Inter-observer agreement (IOA) was performed by an independent examiner for all the ANS measurements. A total of 40 random CBCT images were re-evaluated and excellent agreement was found for all the ANS measurements (average IOA was 0.821). The best IOA value was for *Ax2* (0.912) and the lowest agreement was for *Sag3* (0.730).

## Results

The average age of individuals in our cohort was 40 years. Of the 400 CBCT images, 10 individuals (2.5%) presented with discrepancies in the measurements of ANS between the researchers. Eight of these individuals were from the white population group and 6 of these were males. Table 1 shows the correlations of population group and sex with the different ANS measurements according to age.

**Table 1:** Pearson’s product-moment correlations for population group and sex with age and the ANS measurements

Population group	Age	Sag1	Sag2	Sag3	Ax1	Ax2
Correlation	− 0.332	− 0.396	− 0.263	0.257	− 0.493	0.553
*p*-value	1.018e-11*	2.2e-16*	9.171e-08*	1.81e-07*	2.2e-16*	2.2e-16*
Correlation coefficient	− 0.3316	− 0.3957	− 0.2632	0.2573	− 0.4932	0.5531
Sex	Age	Sag1	Sag2	Sag3	Ax1	Ax2
Correlation	0.095	− 0.072	− 0.391	0.056	− 0.189	0.011
*p*-value	0.06	0.15	4.966e-16*	0.27	< 0.001*	0.83
Correlation coefficient	0.0955	− 0.0722	− 0.3906	0.0558	− 0.1895	0.0107

*p−value statistically significant at *p* <0.001

**Fig. 3:**
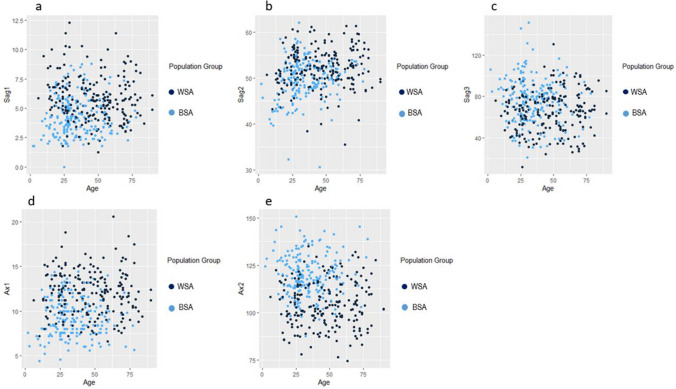
**a–e** Scatterplots showing the relationship of the different ANS measurements for the two population groups according to age. In these scatterplots, light blue indicates BSA individuals and dark blue indicates WSA individuals

The relationship between the two population groups and the different ANS measurements according to age can be visualised in Fig. 3a–e.

**Fig. 4:**
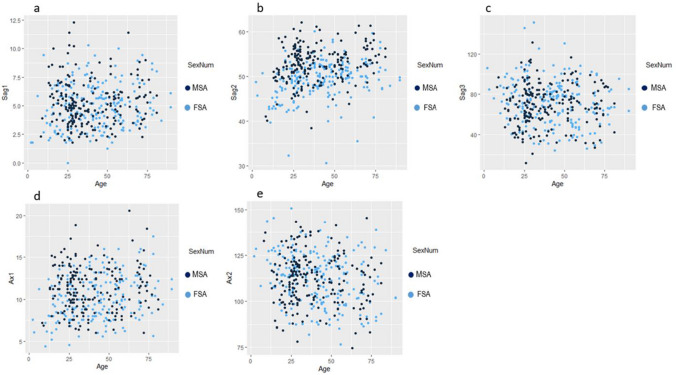
**a–e** Scatterplots showing the relationship of the different ANS measurements for sex and age. In these scatterplots, light blue represents FSA and dark blue represents MSA

The relationship between sex and the different ANS measurements according to age can be visualised in Fig. 4a–e. These included all females and males irrespective of population group.

The data was further divided into separate population and sex groups. Classification decision trees (pruned) were fitted to ascertain the relationship between population group, sex, age and the measurements (*Sag1*, *Sag2*, *Sag3*, *Ax1*, *Ax2*). We further performed a 100-fold cross-validation with training data of size 300 and testing data of size 100. The analysis was conducted in R v 4.1.3 (R Core Team (2022)) using the *rpart* package [26].

**Fig. 5:**
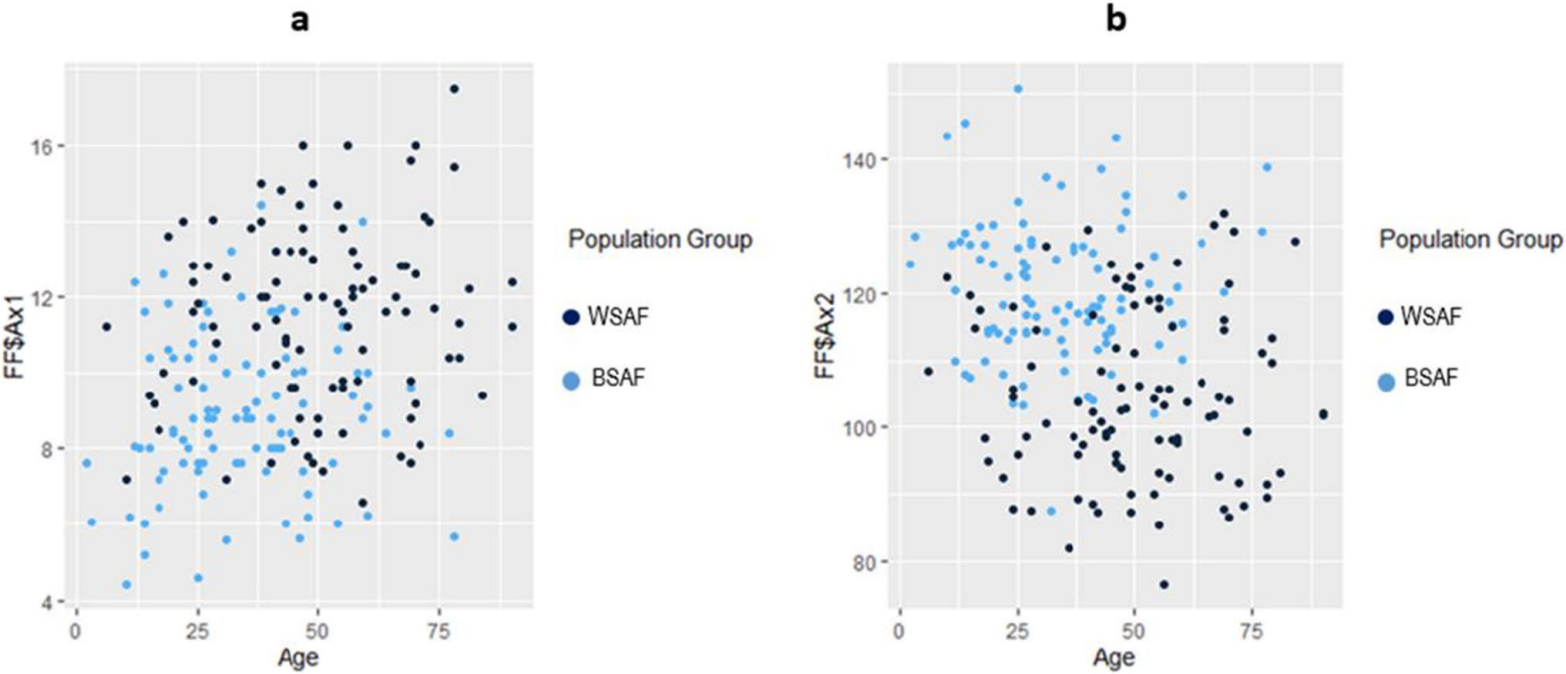
**a and b** Scatterplots for FSA with population group and the axial measurements *Ax1*, *Ax2*. Light blue represents BSAF and dark blue represents WSAF

When *female sex* was considered with population group as the dependent variable, we found that *Ax1* presented with a Pearson correlation that was less than 0.5 (0.4959155) and a *p*-value that was statistically significant (p-value = 8.22e-14). The findings show that when *Ax1* had a measurement of less than 6 mm, this indicated that it was a black South African female (BSAF) (Fig. 5a). Whereas, when *Ax1* was longer than 15 mm, all of the individuals were white South African females (WSAF). Furthermore, in the female population, the *Ax2* angle was found to be statistically significant (*p*-value < 2.2e-16, Pearson correlation of 0.5493407). When females presented with an *Ax2* angle of less than 100°, then the majority of individuals were WSAF and if the angle was greater than 130°, then it was more likely to be a BSAF (Fig. 5b).

**Fig. 6:**
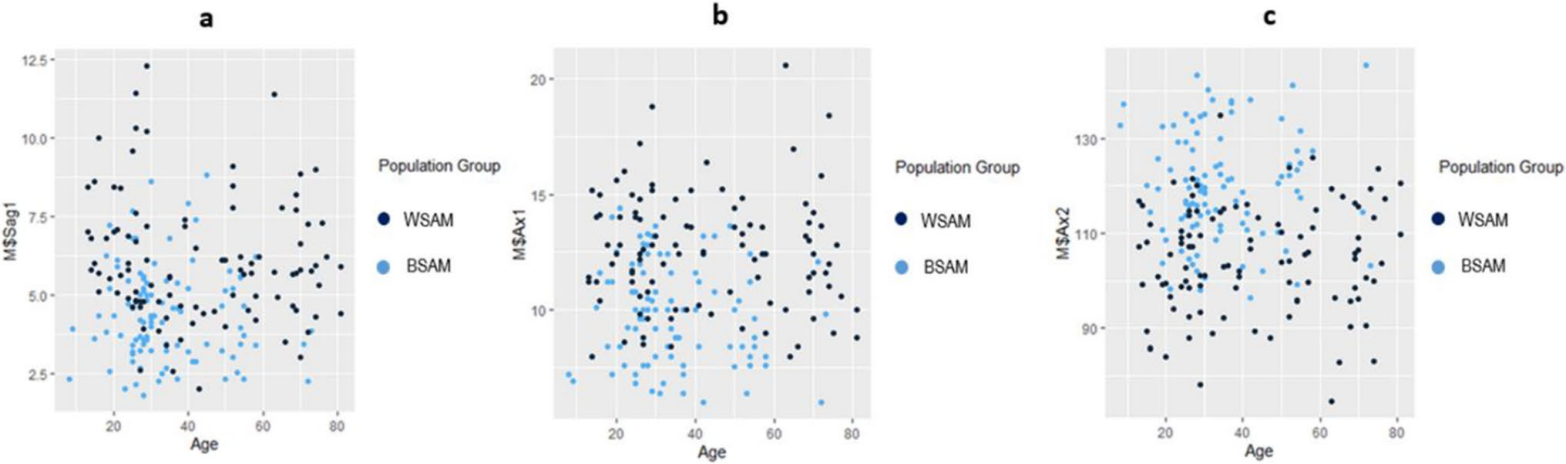
**a–c** Scatterplots for MSA against the population group indicating *Sag1*, *Ax1*, *Ax2* measurements. Light blue represents BSAM and dark blue represents WSAM

In *males*, the *Sag1* measurement showed a Pearson’s correlation of − 0.4389073 and *p*-value of 7.974e-11 with the population group. The findings showed that white South African male (WSAM) individuals were more likely to present with a *Sag1* measurement longer than 9 mm (Fig. 6a), while the majority of individuals that presented with a *Sag1* length shorter than 3 mm were black male South African (BSAM) individuals. A statistically significant association between the length of the ANS (*Ax1*) and the angle of the ANS with the canine bulges (*Ax2*) was seen in males (Pearson’s correlation of − 0.5067143 and 0.5571469 respectively). More BSAM individuals presented with an *Ax1* length of less than 10 mm, with all of individuals that presented with *Ax1* less than 5 mm being BSAM. White South African male (WSAM) individuals presented with a longer *Ax1* and only WSAM presented with an *Ax1* longer than 15 mm (Fig. 6b). Furthermore, the majority of BSAM presented with an *Ax2* angle (Fig. 6c) that was obtuse (larger than 130°) when compared to that of WSAM (100° and smaller). Significantly, individuals that presented with an *Ax2* measurement of less than 90° were strictly WSAM.

A regression decision tree was created for the two population groups with sex as the dependent variable. This was performed using all the ANS variables and separately including and excluding age. When considering the decision trees where age was included, the only ANS measurements that showed significant correlation were *Sag2* and *Ax1* (Root node error = 0.49749).

**Fig. 7:**
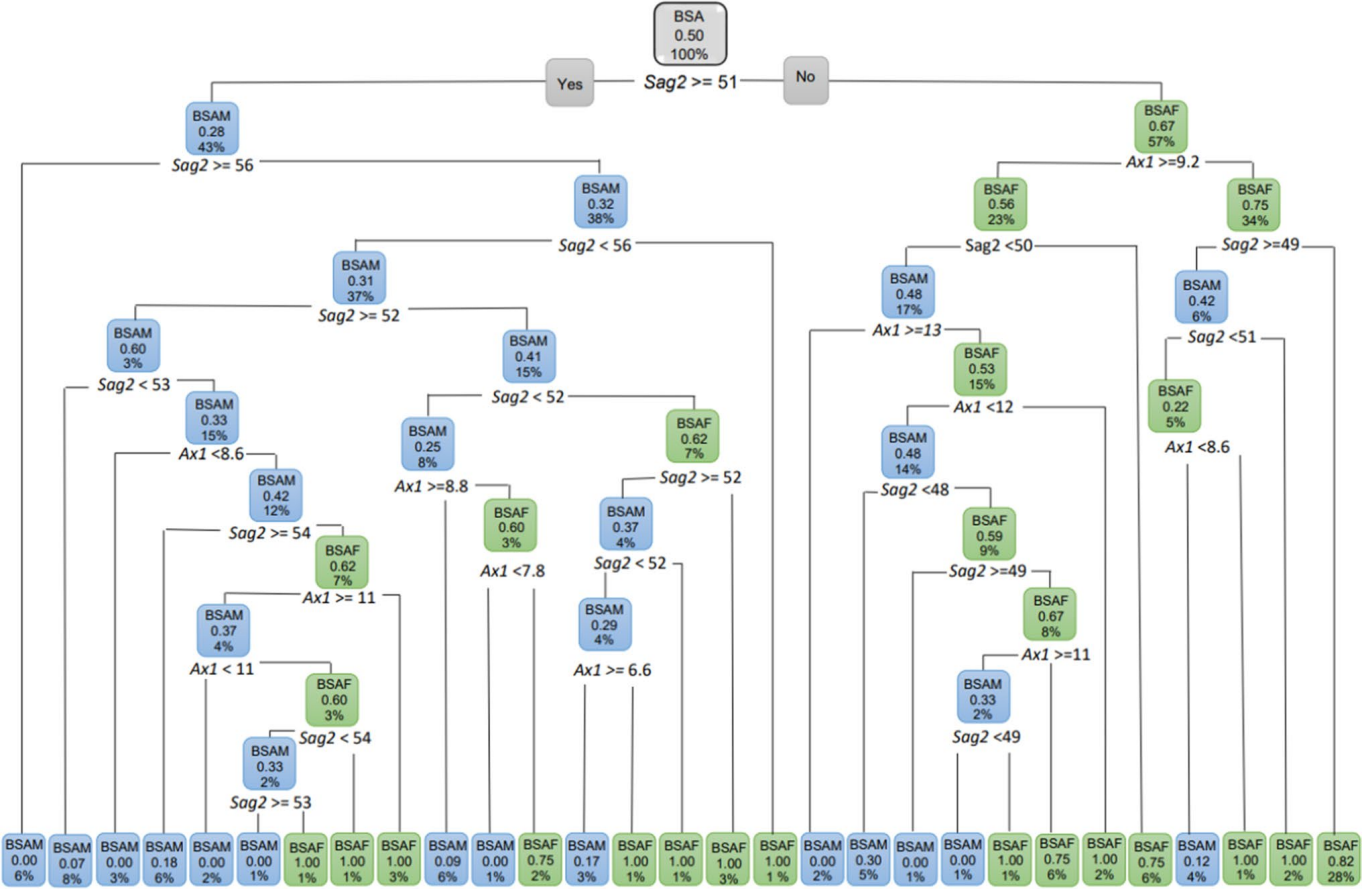
Decision tree for BSA individuals using sex group and ANS measurements *Sag2* and *Ax1.* In this figure green represents BSAF and blue represents BSAM

In BSA individuals (Fig. 7), the *Sag2* measurement was the first measurement to segment the data for sex. When *Sag2* was ≥ 51 mm, 43% of the population was BSAM (with a probability of 28%) and when the *Sag2* measurement was smaller than 51 mm*,* 57% of black population were BSAF (with a probability of 67%). Of this 57% BSAF with a *Sag2* smaller than 51 mm, 34% presented with an *Ax1* that was smaller than 9.2 mm (probability of 75%) and a further 28% of this 34% BSAF had *Sag2* < 49 mm (with a probability of 82%).

**Fig. 8:**
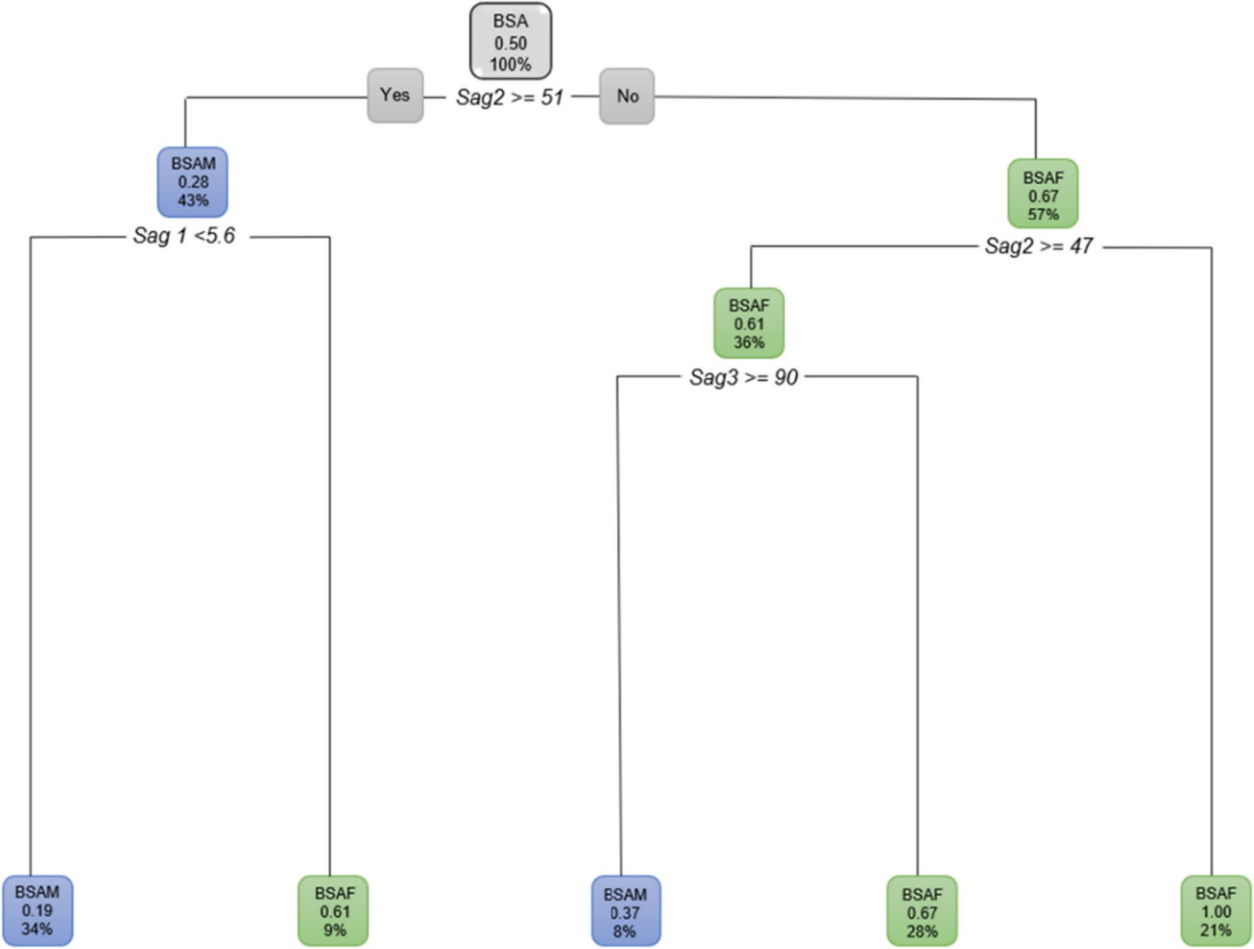
Decision tree for BSA individuals excluding age, using sex group and the sagittal variables only. In this figure green represents BSAF and blue represents BSAM

When BSA individuals were considered excluding age and including all the ANS variables, and when considering only the sagittal variables, *Sag2* ≥ 51 mm remained the first variable to separate the data by sex (Fig. 8). When excluding age and only considering the axial measurements, a similar distribution between BSAM and BSAF was observed with the *Ax1* measurement serving as the first separator of the data.

**Fig. 9:**
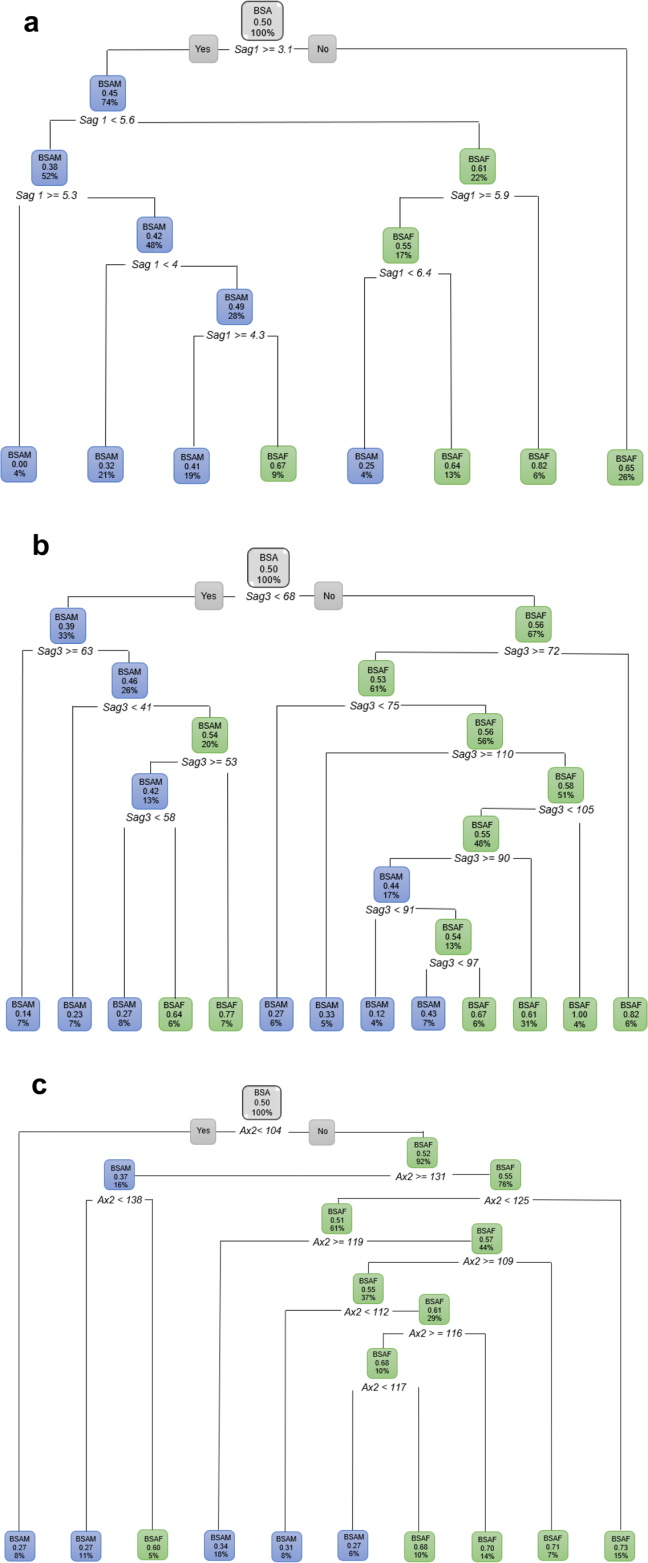
**a** Decision tree for BSA excluding age and only considering *Sag1*. When *Sag1* was 3.1 mm and longer, 74% of the BSA population were BSAM and BSAF (26%) presented with a *Sag1* of less than 3.1 mm. In this figure green represents BSAF and blue represents BSAM. **b** Decision tree for BSA excluding age and only considering *Sag3*. When *Sag3* was larger than 68 °, 67% presented as BSAF and when *Sag3* was smaller than 68 °, 33% were BSAM. In this figure green represents BSAF and blue represents BSAM. **c** Decision tree for BSA excluding age and only considering *Ax2*. When *Ax2* is larger than 104 degrees, 92% were found to be BSAF. In this figure green represents BSAF and blue represents BSAM

When considering the ANS variables independently, *Sag1*, *Sag3* and *Ax2* showed an observable distinction between BSAF and BSAM (Fig. 9a–c).

**Fig. 10:**
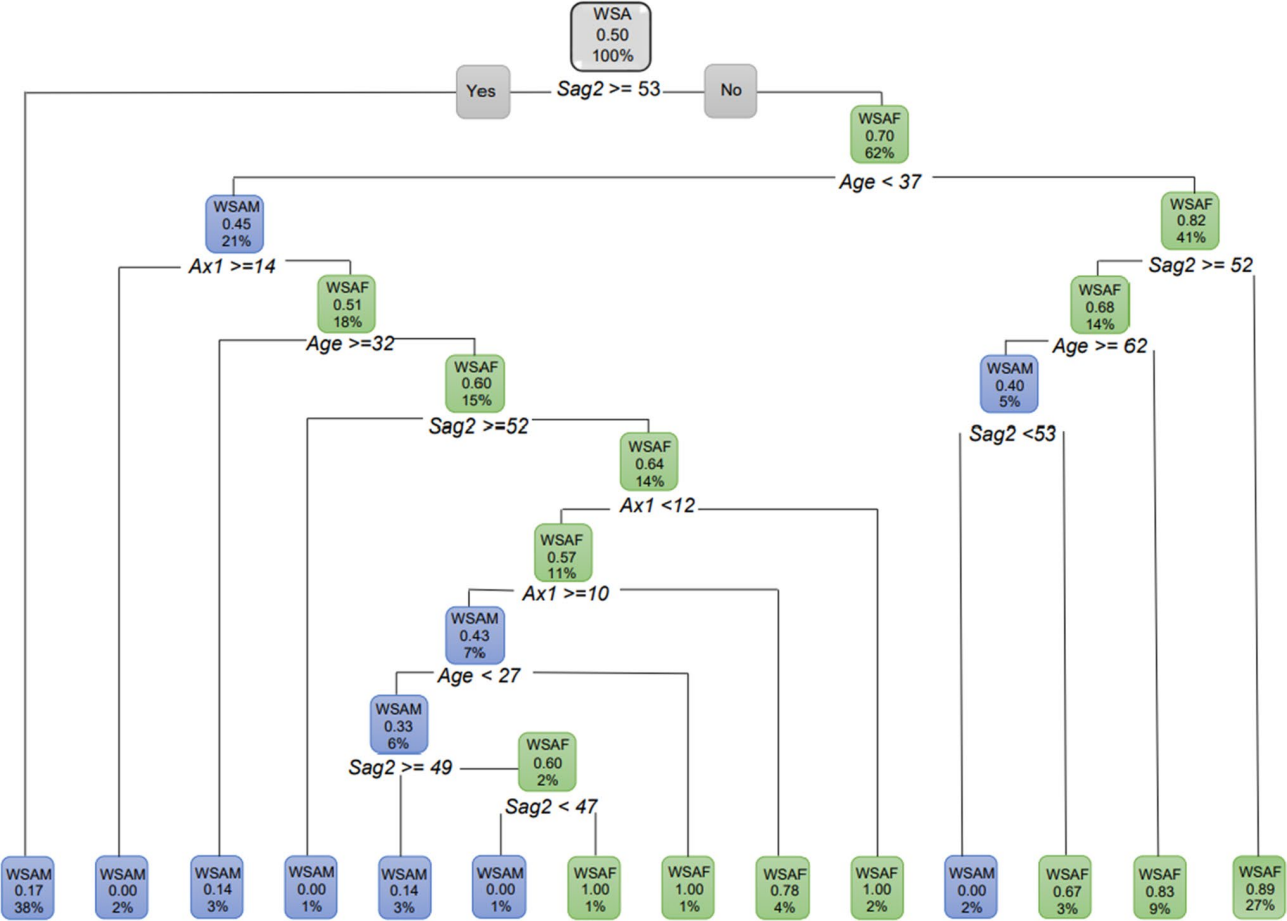
Decision tree for WSA individuals using age, *Sag2* and *Ax1*. In this figure green represents WSAF and blue represents WSAM

The decision tree for WSA individuals with sex as the dependent variable and using age, *Sag2* and *Ax1* (Root node error = 0.49751) showed the following. When the *Sag2* measurement was less than 53 mm, 62% of the individuals were WSAF (with a probability of 70%) and 38% of the WSA had a *Sag2* measurement equal or larger than 53 mm and were WSAM. Of the 62% WSAF that presented with *Sag2* less than 53 mm, 41% of them were older than 37 years (with a probability of 82%). Of these WSAF that were older than 37 years, 27% had a *Sag2* of smaller than 52 mm (with a probability of 89%). Of the WSAF older than 37 years, 14% had a *Sag2* 52 mm and greater. Of these WSAF, 9% were younger than 62 years (Fig. 10).

**Fig. 11:**
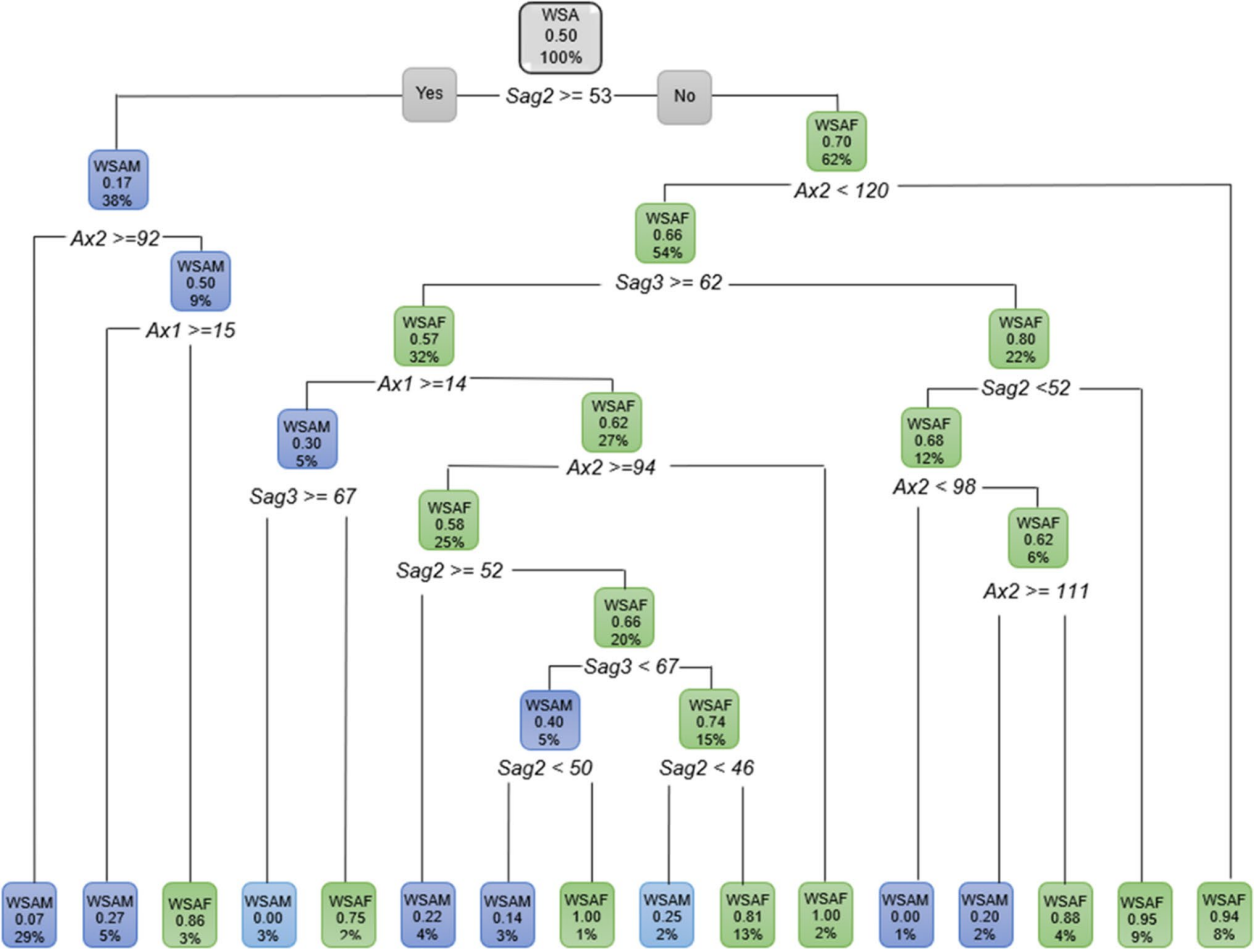
Decision tree for WSA individuals excluding age, using sex and all the ANS measurements. In this figure green represents WSAF and blue represents WSAM

The decision tree for WSA individuals when age was excluded still showed *Sag2* to be relevant in addition to all the other ANS measurements. With age excluded, 62% were WSAF when *Sag2* was less than 53 mm. A further 54% of these WSAF presented with an *Ax2* less than 120° and a further 32% of these has a *Sag3* equal or larger than 62°. WSAM (38%) on the other hand presented with *Sag2* 53 mm and larger, and 29% of these WSAM had an *Ax2* of 92° and greater (Fig. 11).

**Fig. 12:**
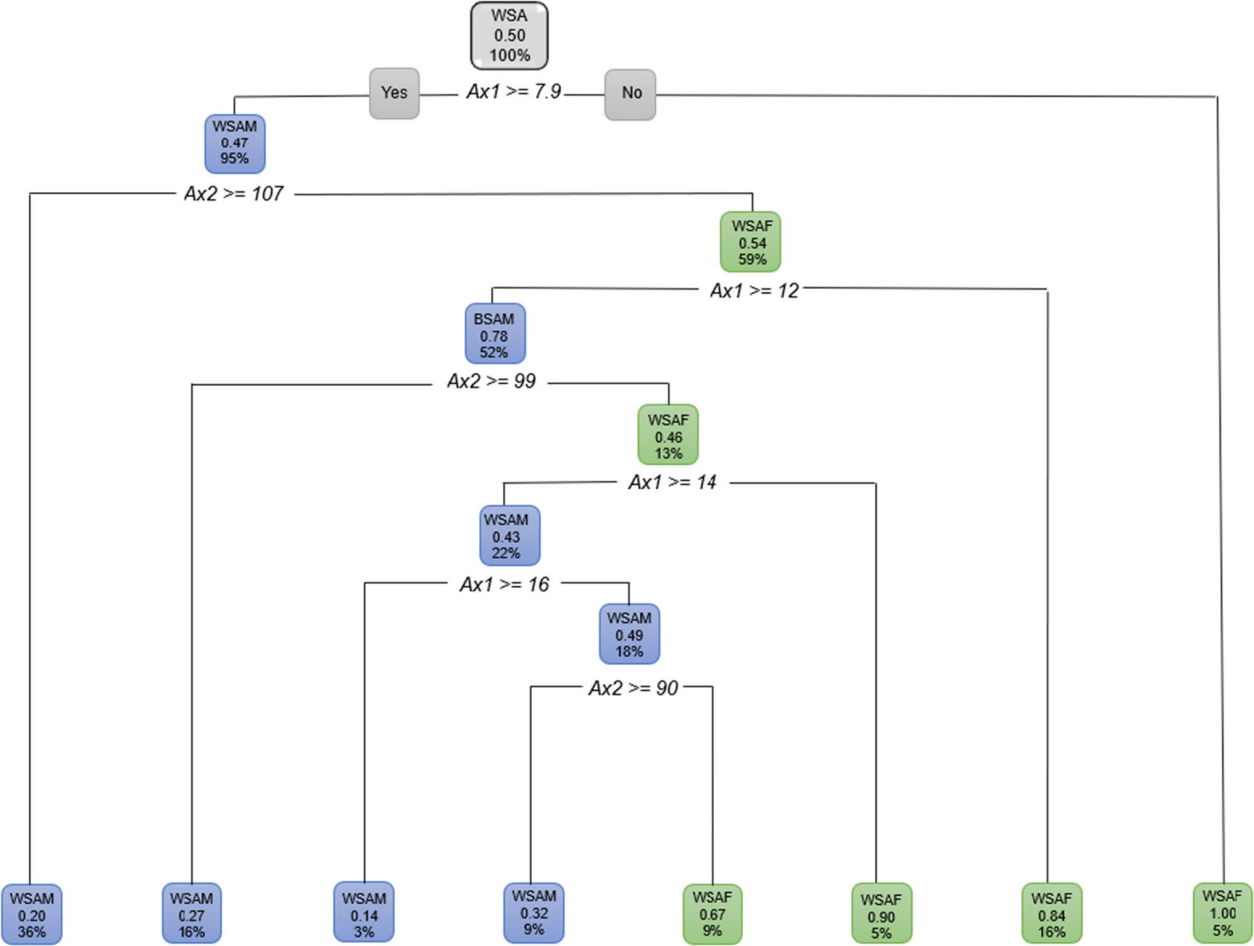
Decision tree for WSA individuals excluding age, using sex and the axial ANS measurements only. In this figure green represents WSAF and blue represents WSAM

When only the sagittal ANS measurements were considered, *Sag2* was still the most prominent measurement in discriminating sex. When only the axial ANS measurements were considered, *Ax1* was found to be 7.9 mm or longer in 95% of WSAM a further 36% of these males presented with an *Ax2* 107° and larger. Only 5% of WSAF presented with *Ax1* smaller than 7.9 mm (Fig. 12).

**Fig. 13:**
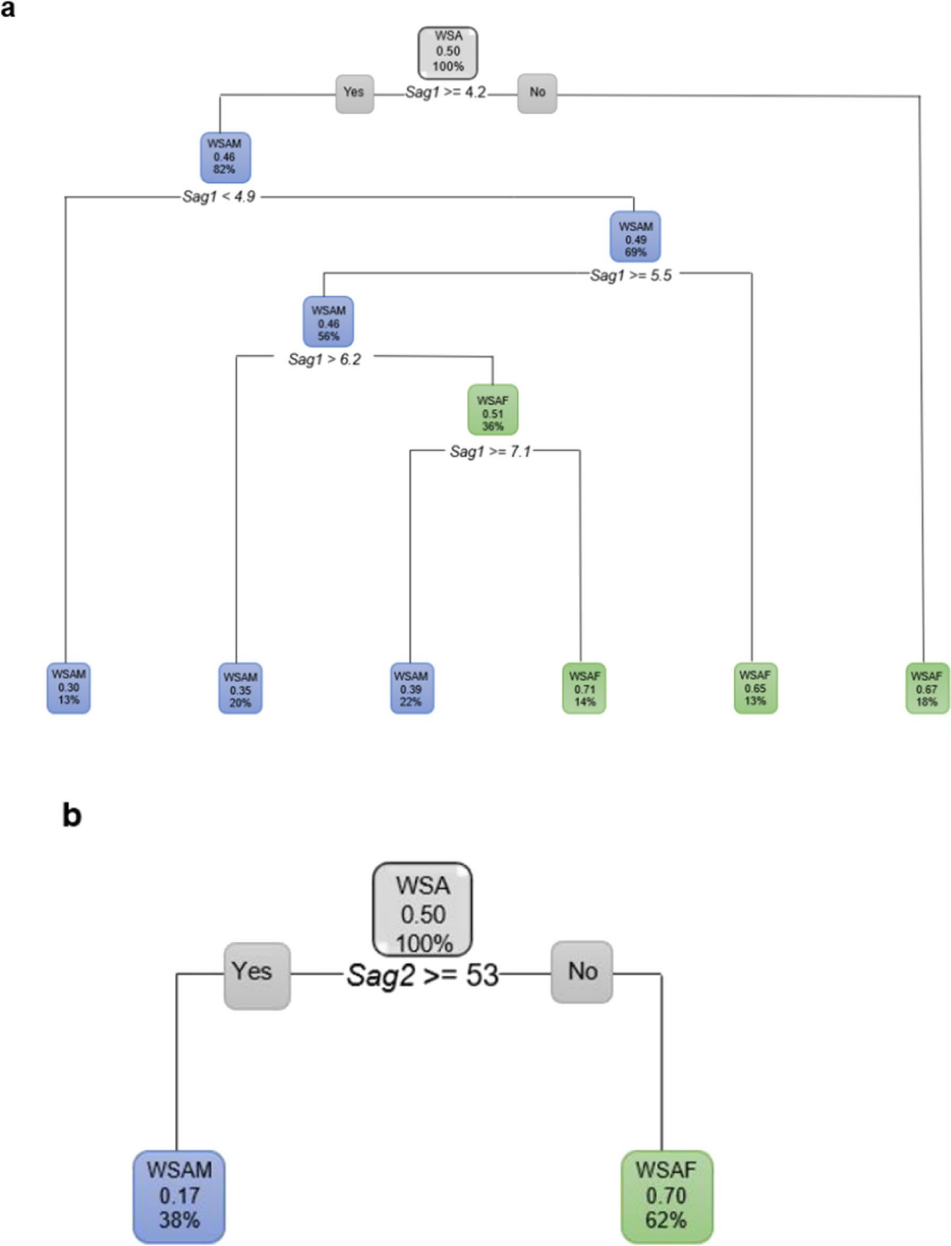

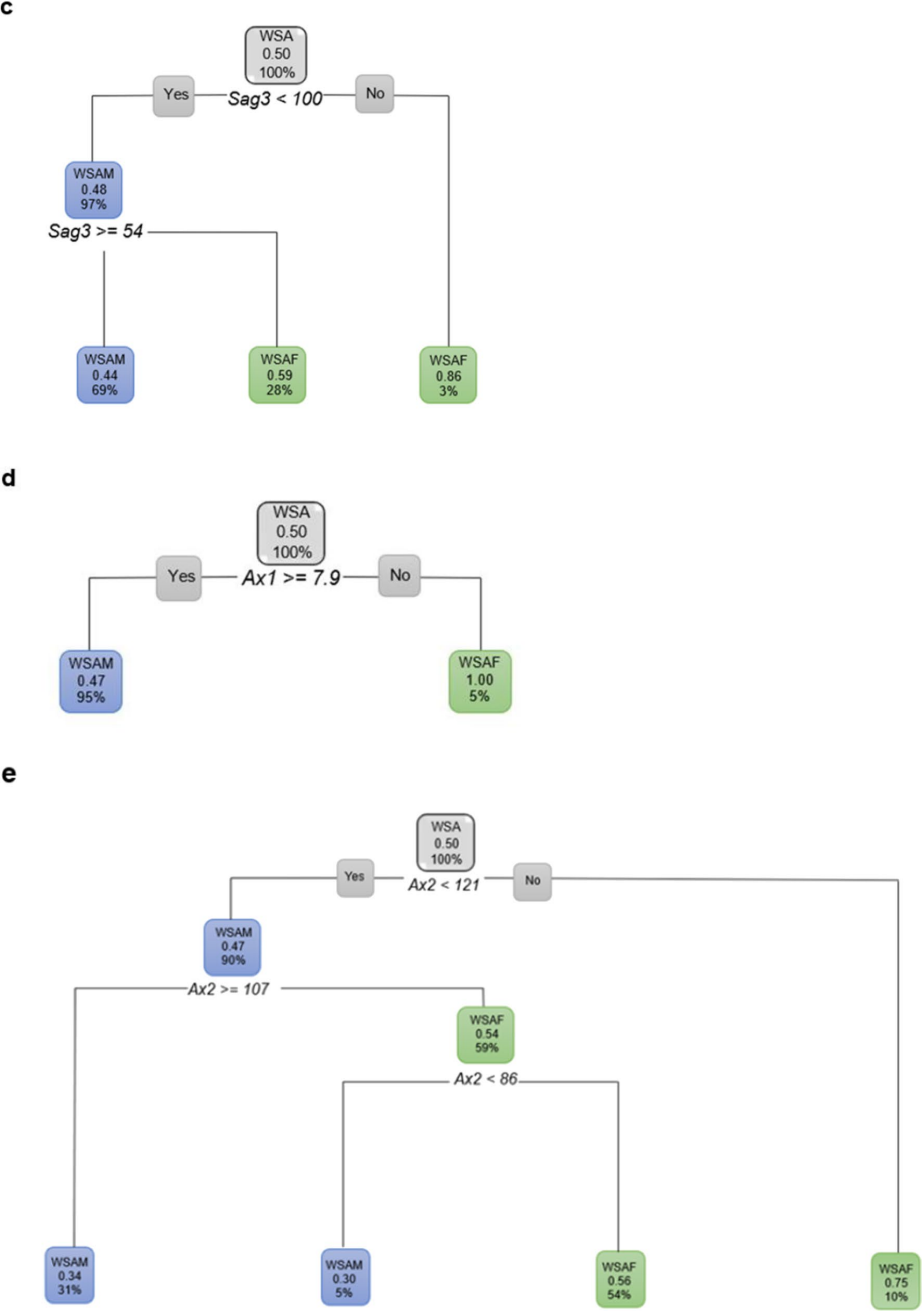
**a** Decision tree for WSA excluding age and including only *Sag1*. When *Sag1* was 4.2 mm or larger, 82% of the WSA population presented as WSAM. When *Sag1* was less than 4.2 mm, 18% of the WSA population presented as WSAF. **b** Decision tree for WSA excluding age and including only *Sag2*. When *Sag2* is 53 mm and longer, only 38% of the WSA population presented as WSAM. When *Sag2* was less than 53 mm, 62% of the WSA population presented as WSAF. **c** Decision tree for WSA excluding age and including only *Sag3*. When *Sag3* is less than 100 °, 97% of the WSA population presented as WSAM. When *Sag3* was more than 100 °, 3% of the WSA population presented as WSAF. **d** Decision tree for WSA excluding age and including only *Ax1*. When *Ax1* is 7.9 mm and larger, 95% of the WSA population presented as WSAM. **e** Decision tree for WSA excluding age and including only *Ax2*. When *Ax2* was less than 121 °, 90% of the WSA population presented as WSAM

When considering the ANS measurements for WSA independently excluding age, all the ANS measurements showed observables differences for sex (Fig. 13a–e).

**Fig. 14:**
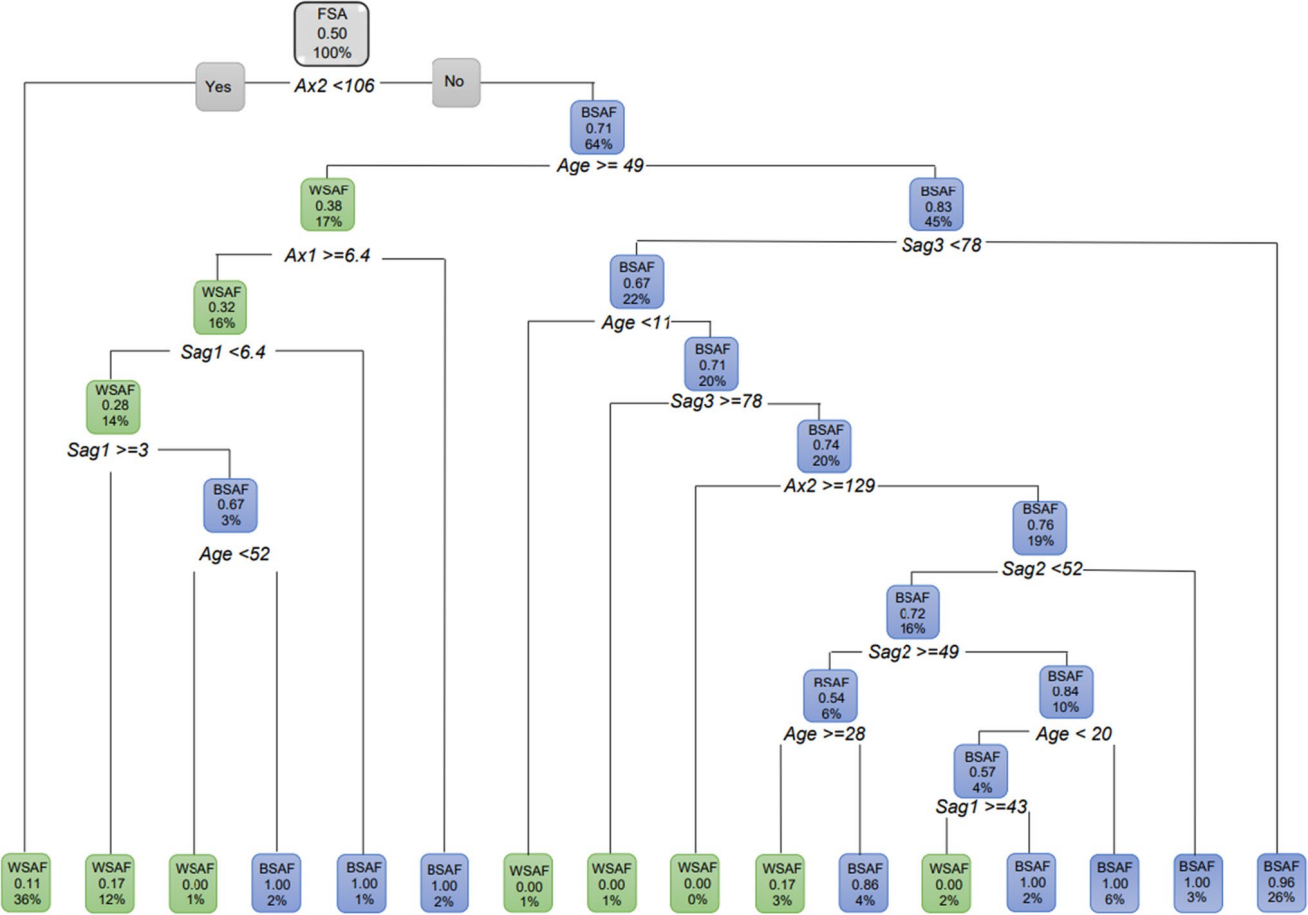
Decision tree for FSA including age and all the ANS measurements. In this figure green represents WSAF and blue represents BSAF

Regression decision trees were created for the two sexes with population as the dependent variable. All correlations were found to be significant for FSA (Root node error = 0.5). When the *Ax2* was larger than 106°, 64% of FSA were BSAF (probability of 71%) whereas 36% of were WSAF when the *Ax2* was smaller than 106° (Fig. 14). Of the 64% BSAF with an *Ax2* of greater than 106°, 45% of them were younger than 49 years. Of these BSAF younger than 49 years, 26% presented with a *Sag3* greater than 78° (with a probability of 96%) and 22% had a *Sag3* less than 78°. Of the 22% BSAF that had a *Sag3* smaller than 78°, 20% (with a 71% probability) were older than 11 years of age. Of the WSAF that were 49 years and older, 17% presented with *Ax1* that was ≥ 6.4 mm.

**Fig. 15:**
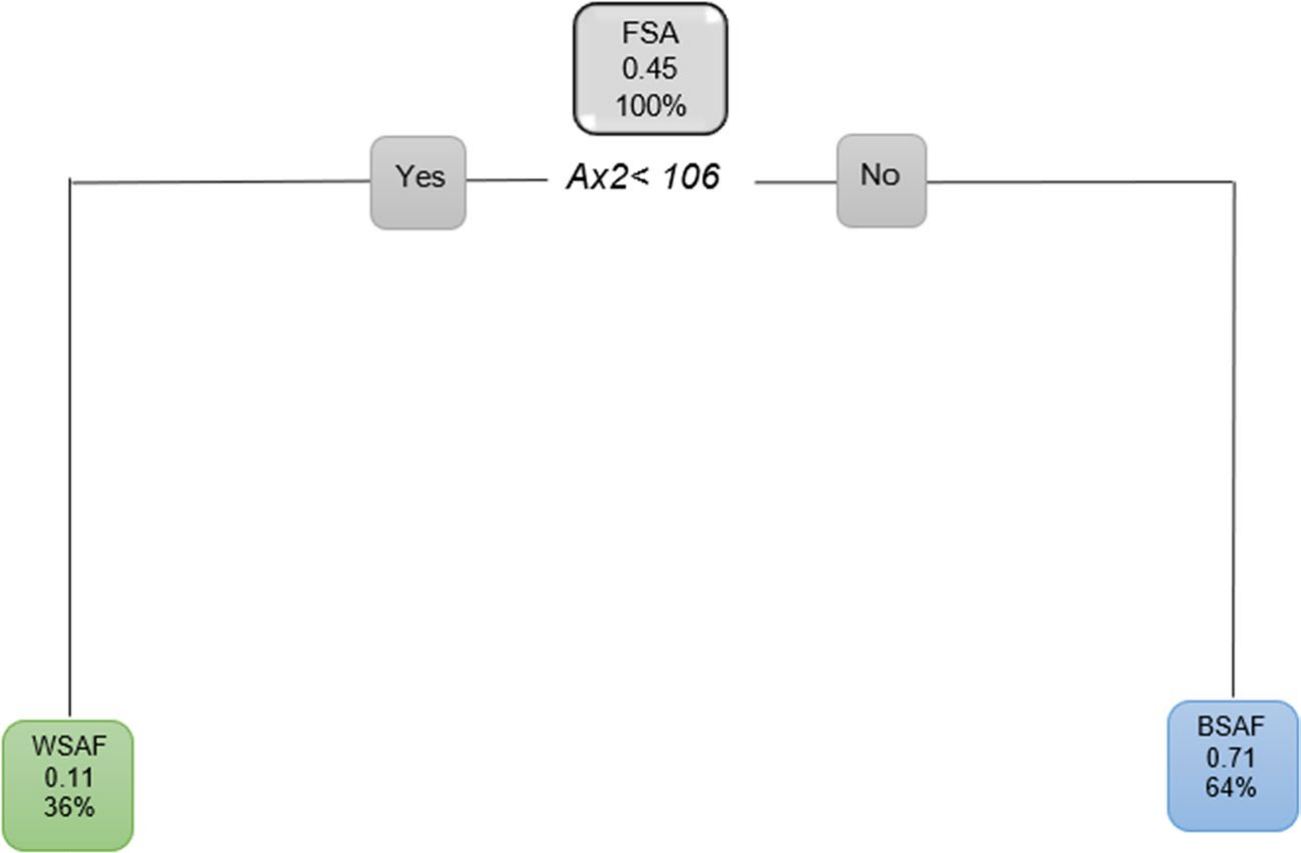
Pruned decision tree for FSA excluding age and *Ax2*

When looking at FSA excluding age for both population groups and all the ANS measurements, the findings were similar to when age was included. When *Ax2* was larger than 106 °, 64% of the FSA population was found to be BSAF (Fig. 15).

**Fig. 16:**
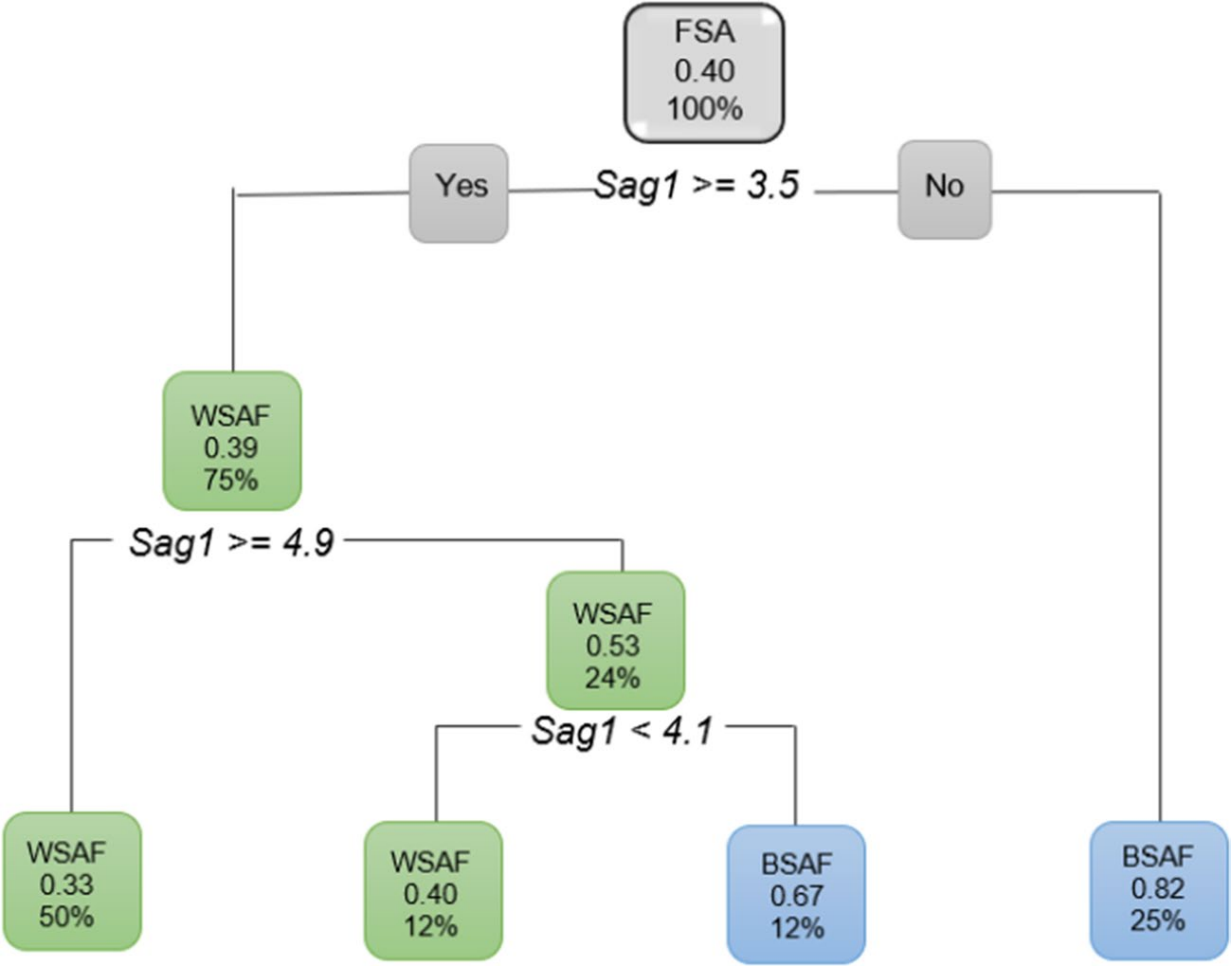
Pruned decision tree of FSA, excluding age and only considering *Sag1*. WSAF (75%) presented with *Sag1* 3.5 mm and larger

When only the sagittal ANS measurements were considered, *Sag1* was the first measurement to separate the data (Fig. 16). *Sag2* and *Sag3* did not show any large differences between the population groups.

**Fig. 17:**
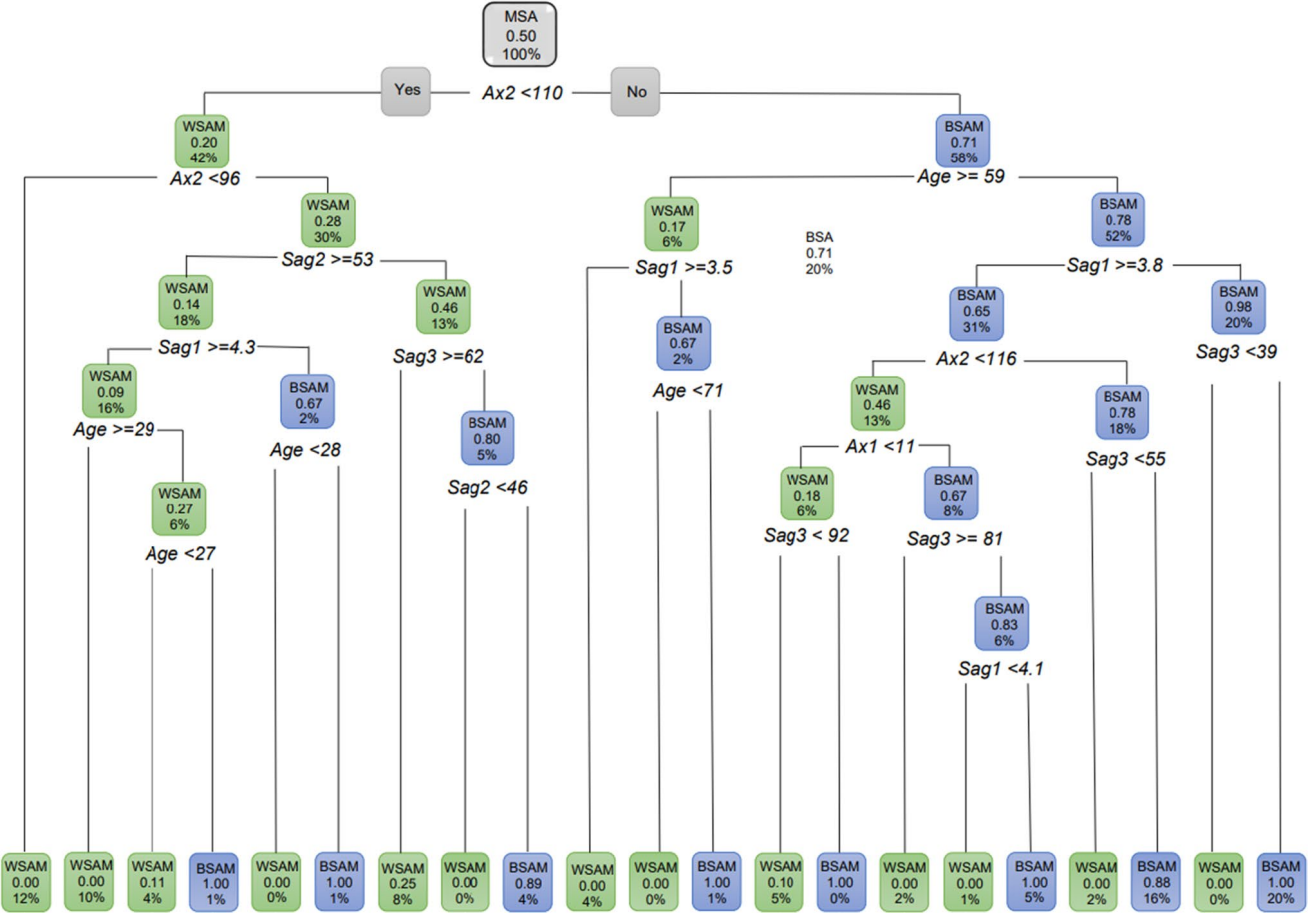
Decision tree for MSA including age and all the ANS measurements. In this image green indicates WSAM and blue indicated BSAM

When considering MSA with population group as the dependent variable and age was included, all correlations were found to be significant (Root node error = 0.495) when performed individually. For MSA, 58% were found to be BSAM when the *Ax2* angle was 110° or larger (probability of 71%) and 42% were found to be WSAM when *Ax2* was less than 110° (Fig. 17). Of the BSAM males that presented with *Ax2* 110° or larger, 52% were younger than 59 years (probability of 78%). In BSAM younger than 59 years, 31% had a *Sag1* length of 3.8 mm or longer. Furthermore, 18% of these BSAM presented with an *Ax2* larger than 116° (probability of 78%) and 16% of these additionally had a *Sag3* angle greater than 55°. The WSAM on the other hand made up the other 42% of the MSA and presented with *Ax2* smaller than 110°. Thirty percent of these WSAM had an *Ax2* larger than 96° (with a probability of 28%). Of these WSAM with an *Ax2* larger than 96°, 18% further presented with a *Sag2* 53 mm or longer. Of these WSAM, 16% had a *Sag1* of 4.3 mm and greater and 10% of these were older than 29 years.

**Fig. 18:**
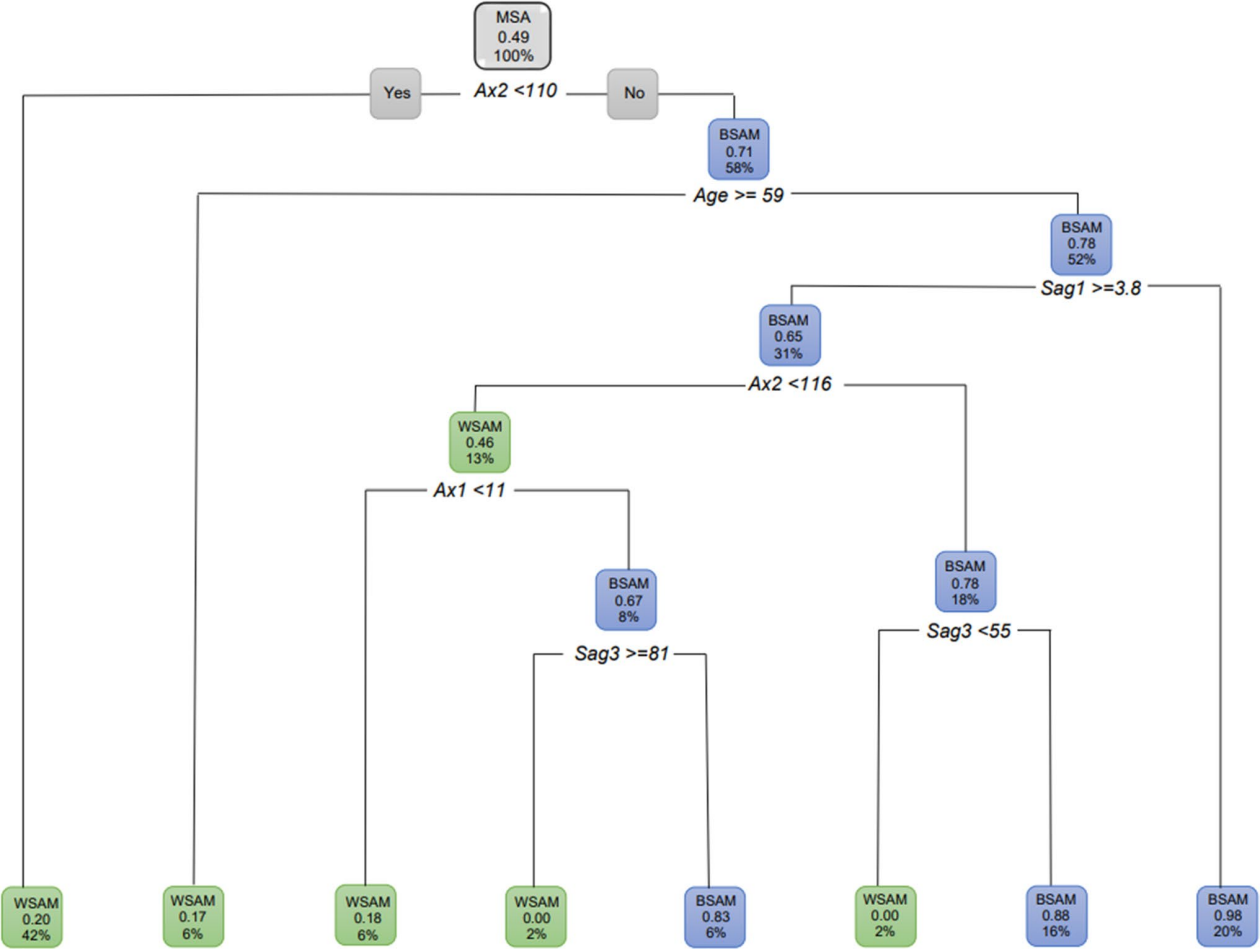
Decision tree for MSA including age with population group with *Sag2* removed. In this image green indicates WSAM and blue indicated BSAM

However, when the classification tree was fitted for MSA, *Sag2* was not found to be significant. When *Sag2* was then removed, the following was found for MSA (Fig. 18). Fifty eight percent of the MSA that presented with *Ax2* larger than 110° (71% probability) were BSAM. Of these BSAM, 52% were younger than 59 years of age and 31% of these BSAM younger than 59 years had a *Sag1* 3.8 mm and longer. Eighteen percent of these BSAM further presented with an *Ax2* larger than 116° (65% probability).

**Fig. 19:**
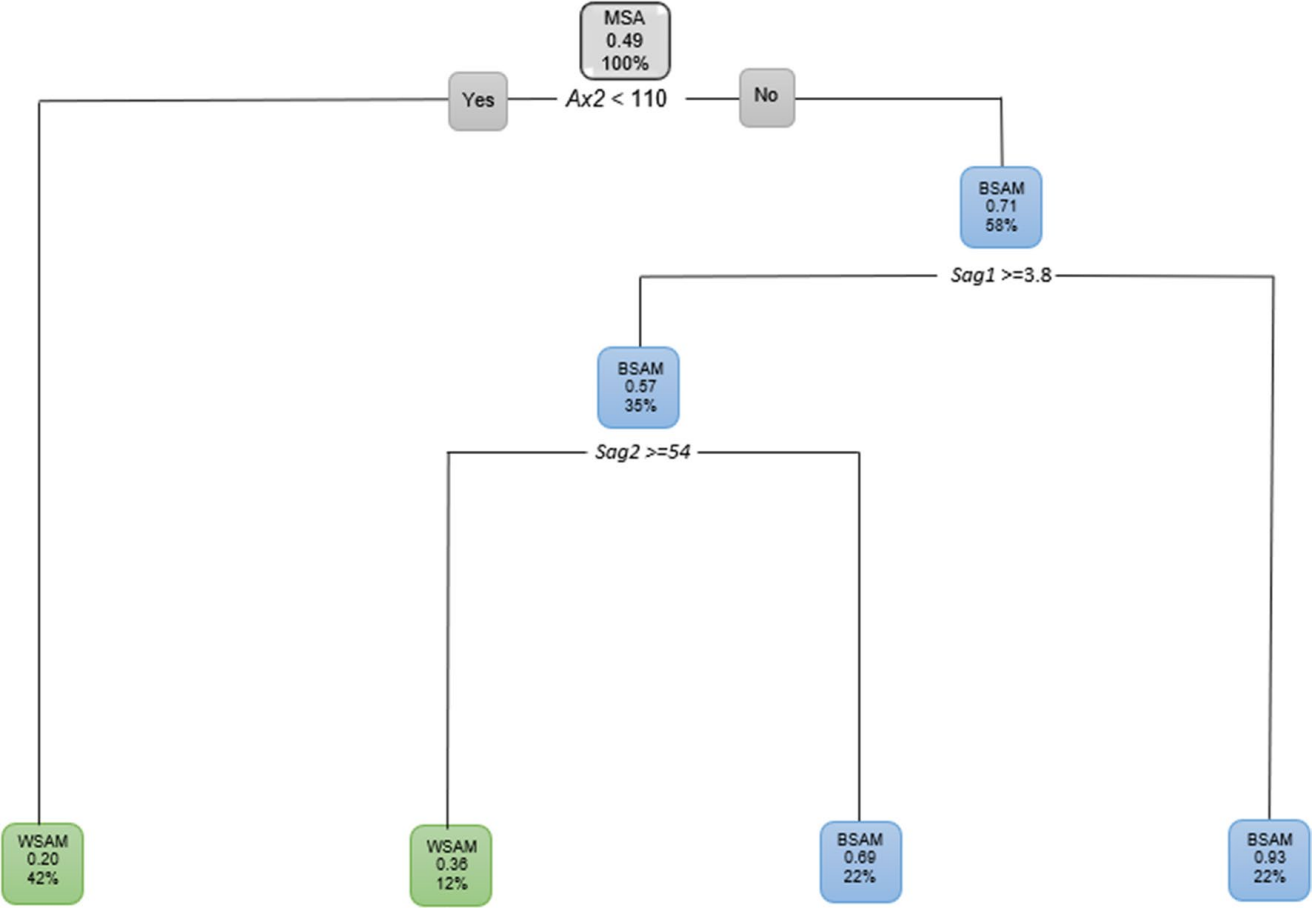
Decision tree for MSA with age excluded for population group and all the ANS measurements. In this image green indicates WSAM and blue indicated BSAM

When age was excluded for MSA and all the ANS measurements included, 42% of the population were WSAM when *Ax2* was less than 110 °. The remainder of the male population (58%) presented with an *Ax2* larger than 110 ° and were BSAM. Of the 58% BSAM, 35% presented with a *Sag1* 4.3 mm and larger (Fig. 19).

**Fig. 20:**
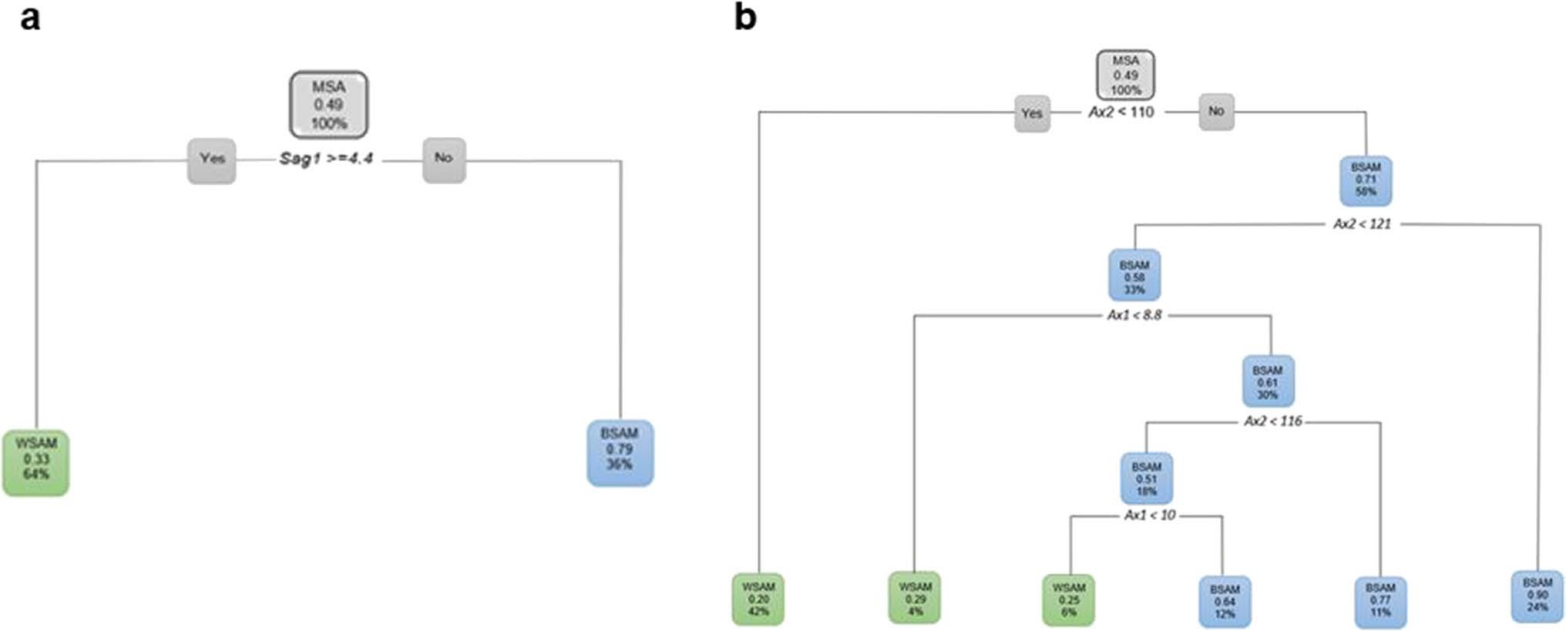
**a and b** Decision trees for MSA excluding age for population group and the sagittal and axial ANS measurements

**Fig. 21:**
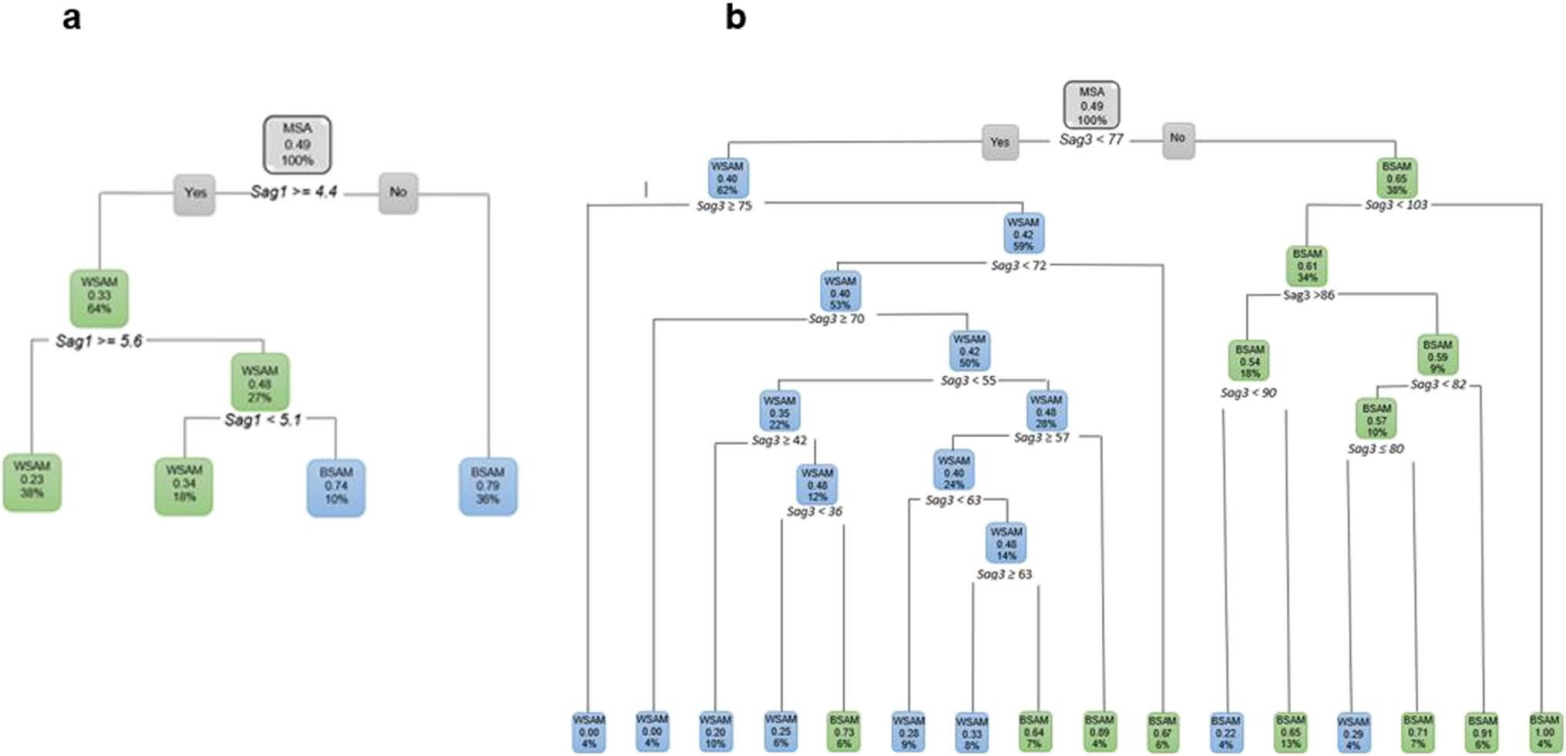
**a and b** Decision trees for MSA excluding age for population group and *Sag1* where 64% WSAM presented with Sag1 of 4.4 mm and longer and *Sag3 *larger than 77 °

When only considering the sagittal variables, *Sag1* was the first measurement to separate the data with 64% of MSA as WSAM when *Sag1* ≥ 4.4 (Fig. 20a). When only the axial variables were considered, 58% of MSA presented as BSAM when *Ax2* was larger than 110 ° (Fig. 20b). When considering the axial and sagittal variables independently, both *Sag1* and *Sag3* were relevant in separating WSAM and BSAM (Fig. 21). *Sag2*, *Ax1* and Ax2 were fairly equally distributed in the black and white male populations

## Discussion

In this study, the metric data was separated using fitted classification decision trees which were pruned to ascertain the relationship between population group, sex and age with the ANS measurements (*Sag1*, *Sag2*, *Sag3*, *Ax1*, *Ax2*). A decision tree is a visual representation of the non-parametric data using a tree-like system. It begins with a root node that is used to predict the data, which then ends as terminal nodes. From the first node, called the root node, the data branches for each possible outcome leading to what is referred to as decision nodes. This results in the formation of other decision nodes separating the data in a manner that eventually ends in terminal nodes. An essential practice in creating a decision tree is that of pruning which eliminates non-fundamental sub-nodes. The construction of an ideal decision tree depends upon the specific algorithm used [27].

Metric data on the ANS is scarce and this is one of the first studies to investigate the morphology of the ANS using measurements on CBCT images in different population groups and sexes. The ANS is both clinically and anthropologically relevant. The clinical relevance relates to is function in dictating the morphology of the human face in the area of the lower portion of the nose and upper lip [2]. Most research articles in this regard are related to augmentation rhinoplasty [2, 28, 29]. Even in the surgical context, reference is made to the differences of cranial and facial morphology in different population and sex groups [28, 30].

Forensic identification can be applied in both living and deceased individuals as well as for the identification of human remains. Different methodologies can be used as part of the identification process. These include fingerprints, genetic fingerprinting and DNA profiling, forensic anthropology, forensic dentistry and marked orthopaedic/dental devices [31–38]. Furthermore, forensic anthropologists can use the morphology and features of the skeletal bones to assess for both population and sex [39–43]. Population specific standards for South African individuals are important, highlighted by the low accuracy when applying certain classifications [15, 19]. A study on non-metric cranial traits within three South African population groups found that Black and Coloured South African individuals were often classified as either African or Asian with regards to nasal bone contour, the inferior nasal margin and the anterior nasal spine whereas White South African individuals predominantly exhibited European characteristics for these cranial traits. In this study, almost all the cranial variables were significant for population [12]. Estimation of population is linked to sex and for this reason sex determination is often the first step in the identification process [44]. A South African study by Işcan and Steyn showed that the crania are more discriminatory than the mandibles especially in females [45]. Combing metric analysis and osteology improves accuracy of sex identification [46]. The ability to estimate the sex and population of an individual aids in creating a biological profile and can narrow the search for individuals in the identification process.

In this study, ANS measurements demonstrated significant statistical associations with FSA when population group was the dependent variable, even when accounting for age. However, in the case of MSA, while individual correlations revealed significant associations for all ANS measurements, the classification tree analysis identified *Sag2* as non-significant. These findings indicate that WSA individuals exhibited longer ANS measurements, resulting in a more acute angle, while BSA individuals had shorter ANS measurements and a more obtuse angle. These differences are likely attributed to variations in mid-facial morphology between the population groups, consistent with findings reported by Mooney & Siegel [10], who also observed more prominent ANS measurements in white skull specimens compared to black specimens. Additionally, existing literature suggests that climatic differences may contribute to variations in mid-facial morphology, particularly in the nasal region, with populations from tropical climates showing wider nasal apertures and those from temperate regions displaying narrower nasal apertures [47–49].

When considering sex, two ANS measurements (*Sag2* and *Ax1*) were found to be statistically significant when age was both included and excluded. This indicates that sex differences in both BSA and WSA individuals did not differ extensively. Furthermore, age was additionally found to be significant for WSA when age was included in the decision tree. A small difference in the length of the ANS (*Ax1*) was noted for both sex and population groups in this study. WSAF presented with an *Ax1* longer than 12 mm whereas WSAM had an *Ax1* 15 mm or longer. BSAF presented with an *Ax1* 8 mm or shorter whereas BSAM had an *Ax1* less than 10 mm. Overall WSA individuals presented with longer ANS length and BSA individuals had a shorter ANS length. The angle of the ANS (*Ax2*) correlates to the length of the ANS (*Ax1)*, as the shorter the length of the ANS, the more obtuse the angle that will, be formed from the anterior point of the ANS to the canine bulges. The minimal differences in the length and angle of the ANS can be attributed to sexual dimorphism.

When age was excluded and the ANS measurements considered independently, in WSA individuals, *Sag3* showed large differences between WSAM and WSAF with 97% of WSAM presenting with *Sag3* less than 100 ° and *Sag2* (less than 52 mm) and *Sag1* (less than 4.2 mm) distinguished WSAF from WSAM. Prior studies have also identified variations in facial morphology among different population groups and sexes. For instance, Talbert et al. [50] observed differences between African and Caucasian American males and females. African American females displayed broader faces, wider nasal bases, and more prominent lips compared to their Caucasian American counterparts. Similarly, African American males had wider nasal bases and more prominent lips when compared to Caucasian American males. Another study of individuals mainly of Iraqi ancestry revealed prominent ANS length [51].

This study aimed to assess ANS metric variations among black and white, male and female individuals in South Africa. Such radiographic differences could have practical applications in forensic identification, particularly when assessing deceased individuals without accessible external bodily features or skeletal remains. Advancements in autopsy techniques, shifting from invasive methods to digital autopsies utilizing full-body radiography, may open doors for using cranial features and ANS measurements in identification [43, 52, 53]. Nevertheless, the limited availability of metric data for the ANS in diverse populations and sexes necessitates further research before considering its widespread use.

Damage or fracture to the nasal region and changes to the surrounding bony structures as in edentulous patients or patients with missing teeth in the canine region could alter the morphology and therefore the measurements of the ANS. In this study, we did not exclude edentulous patients or patients with missing teeth. The extent of the bony changes depending on the clinical scenario could impact on the use of the ANS measurements in identification [54–56]. Bone resorption after tooth loss does result in alveolar bone resorption [57, 58] but the extent of the number of teeth lost and the time that the teeth have been missing on the ANS still requires further investigation. Other instances, like harvesting of the bone of the ANS to treat bone loss around implants or surgical procedures in the midfacial region [59–61], will also alter the anatomy and render the ANS of no value for identification.

## Limitations

The limitations of the study include the fact that the ANS is a bony structure that can be altered in instances of tooth loss, trauma or surgery. This would impact on the usability of this structure in these individuals. This study did not exclude individuals with missing teeth or an edentulous maxilla. The influence of tooth loss or surgery, especially in the anterior maxilla should also be evaluated in future studies.

## Conclusions

The ability to estimate the sex and population of an individual aids in creating a biological profile and can narrow the search for individuals in the identification process. The ANS could provide discriminatory information in the identification process, especially with advancements in using post mortem radiology and in scenarios where external and soft tissue information has been lost. This is the first study to investigate a larger sample size in the metric analysis of the ANS on CBCT. We showed that WSA individuals presented with a longer ANS that produced a more acute angle whereas BSA individuals presented with a shorter ANS and a more obtuse angle. All the ANS measurements showed statistical significance for FSA in determining population group. In MSA, all the ANS measurements were significant individually, but when fitted to the classification tree for MSA, *Sag2* did not show any significance. When considering sex, 2 of the ANS measurements (*Sag2* and *Ax1*) were significant and age was additionally found to be significant in WSA. Thereby implying that the ANS is more relevant for population discernment. Ancestral differences were found for ANS measurements and emphasize the need for population specific data. To achieve an accurate and reliable identification the results from this study should be combined with other indicators. The evaluation of the ANS by using CBCT images and measurements could be a valuable additional tool in the identification process.

## Data Availability

All data can be made available if required.
